# Transcriptome analysis of carbohydrate metabolism during bulblet formation and development in *Lilium davidii* var. *unicolor*

**DOI:** 10.1186/s12870-014-0358-4

**Published:** 2014-12-19

**Authors:** XueYan Li, ChunXia Wang, JinYun Cheng, Jing Zhang, Jaime A Teixeira da Silva, XiaoYu Liu, Xin Duan, TianLai Li, HongMei Sun

**Affiliations:** College of Horticulture, Shenyang Agricultural University, Shenyang, Liaoning 110866 P R China; ᅟ, P. O. Box 7, Miki-cho post office, Ikenobe 3011-2, Kagawa-ken, 761-0799 Japan

**Keywords:** *Lilium*, Bulblet formation and development, Transcriptome, Starch and sucrose metabolism, Gene expression file

## Abstract

**Background:**

The formation and development of bulblets are crucial to the *Lilium* genus since these processes are closely related to carbohydrate metabolism, especially to starch and sucrose metabolism. However, little is known about the transcriptional regulation of both processes. To gain insight into carbohydrate-related genes involved in bulblet formation and development, we conducted comparative transcriptome profiling of *Lilium davidii* var. *unicolor* bulblets at 0 d, 15 d (bulblets emerged) and 35 d (bulblets formed a basic shape with three or four scales) after scale propagation.

**Results:**

Analysis of the transcriptome revealed that a total of 52,901 unigenes with an average sequence size of 630 bp were generated. Based on Clusters of Orthologous Groups (COG) analysis, 8% of the sequences were attributed to carbohydrate transport and metabolism. The results of KEGG pathway enrichment analysis showed that starch and sucrose metabolism constituted the predominant pathway among the three library pairs. The starch content in mother scales and bulblets decreased and increased, respectively, with almost the same trend as sucrose content. Gene expression analysis of the key enzymes in starch and sucrose metabolism suggested that sucrose synthase (SuSy) and invertase (INV), mainly hydrolyzing sucrose, presented higher gene expression in mother scales and bulblets at stages of bulblet appearance and enlargement, while sucrose phosphate synthase (SPS) showed higher expression in bulblets at morphogenesis. The enzymes involved in the starch synthetic direction such as ADPG pyrophosphorylase (AGPase), soluble starch synthase (SSS), starch branching enzyme (SBE) and granule-bound starch synthase (GBSS) showed a decreasing trend in mother scales and higher gene expression in bulblets at bulblet appearance and enlargement stages while the enzyme in the cleavage direction, starch de-branching enzyme (SDBE), showed higher gene expression in mother scales than in bulblets.

**Conclusions:**

An extensive transcriptome analysis of three bulblet development stages contributes considerable novel information to our understanding of carbohydrate metabolism-related genes in *Lilium* at the transcriptional level, and demonstrates the fundamentality of carbohydrate metabolism in bulblet emergence and development at the molecular level. This could facilitate further investigation into the molecular mechanisms underlying these processes in lily and other related species.

**Electronic supplementary material:**

The online version of this article (doi:10.1186/s12870-014-0358-4) contains supplementary material, which is available to authorized users.

## Background

Lilies (*Lilium* spp.), a group of monocotyledonous ornamental plants, are one of the major bulbous flowers in the floriculture industry [1]. Taxonomically, the *Lilium* genus is comprised of 115 species, about 55 of which originate from PR China [2]. *L. davidii* var. *unicolor*, a mutation of *L. davidii* Duchartre, is an important genus, both economically and ornamentally, in Lanzhou, Gansu province in PR China. It is renowned for its large size, white and thick flesh as well as sweet taste [3], and its flaming and flamboyant color provide its ornamental value. Besides, lily scales are a rich source of proteins, carbohydrates, lipids, amino acids, and bioactive compounds such as phenolic glycosides, steroidal saponins and alkaloids [4]. In addition, *L. davidii* var. *unicolor* bulbs, which are considered health food due to their abundant nutritional value (11.46% starch, 10.39% sucrose, 5.61% pectin, 3.36% protein), are used in Chinese medicine in different forms as fresh bulbs, dried scales, as well as powder to treat heart and lung ailments [5].

Reproduction of the *Lilium* genus can be achieved through various approaches, including scale cuttings, bulblets or bulblets on stems, bulbils, seed reproduction and tissue culture [6]. In all cases, the formation and development of bulblets are crucial in the life-cycle of this plant. Despite the wealth of literature on horticultural production and tissue culture of *Lilium*, the genetic mechanisms that govern bulblet development still remain unexplored and unclear. The growth and development of *Lilium* bulbs are closely related to carbohydrate metabolism [7], because carbohydrate can serve as building blocks and as an energy source for development of the photosynthetic apparatus. As the main forms of carbohydrates, starch and sucrose are crucial to the balance and coordination of multiple forms of carbohydrates [8,9]. Starch-sucrose metabolism remains a hot topic in plant physiology and biochemistry [10]. However, starch and sucrose metabolism is a complex physiological process due to its close connection with soluble sugar, with dozens of enzymes involved in carbohydrate metabolism [11].

**Figure 1:**
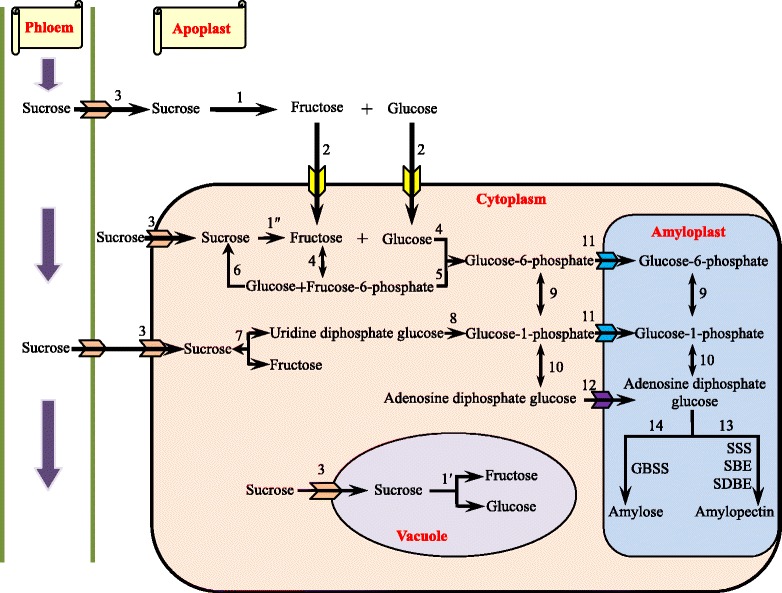
**Sucrose and starch biosynthetic pathway in non**-**photosynthetic cells.** (1) Cell wall invertase (EC 3.2.1.26), (1′) cytoplasmic invertase (EC 3.2.1.26), (1″) vacuolar invertase (EC 3.2.1.26), (2) hexose transporters, (3) sucrose transporters, (4) hexokinase (EC 2.7.1.1), (5) glucose phosphate isomerase (EC 5.3.1.9), (6) sucrose phosphate synthase (EC 2.4.1.14), (7) sucrose synthase (EC 2.4.1.13). (8) UDPG pyrophosphorylase (EC 2.7.7.9), (9) G-6-P by phosphoglucomutase (EC 2.7.5.1), (10) ADPG pyrophosphorylase (EC 2.7.7.27), (11) phosphate translators, (12) ADPG transporter, (13) soluble starch synthase (EC 2.4.1.21), starch branching enzyme (EC 2.4.1.18), starch debranching enzyme (EC 3.2.1.10) (14) granule-bound starch synthase (EC 2.4.1.21). See text for details. (Based on Angeles-Núñez et al. [12-20]).

In the non-photosynthetic cells of higher plants, sucrose, which is transported from the photosynthetic apparatus, is cleaved to its constituent monosaccharides, hexoses or phosphorylated hexoses, which can then be used either in metabolic or biosynthetic reactions [12]. Sucrose is degraded by four different enzymatic mechanisms [13-15]. Firstly, it is hydrolysed into hexoses (glucose and fructose) by cell wall invertase (CWIN, EC 3.2.1.26) in the apoplast (1 in Figure 1). Hexoses generated are then transported into the cytosol by hexose transporters (2 in Figure 1). Secondly, cytosolic sucrose transported from the phloem by sucrose transporters (3 in Figure 1) may also be taken up into vacuoles for hydrolysis by vacuolar invertase (VIN) (1′ in Figure 1). Both the remaining two mechanisms take place in the cytosol. Thirdly, sucrose is hydrolysed into hexoses by cytoplasmic invertase (CIN) (1″ in Figure 1). Hexoses are converted into hexose-6-phosphates by hexokinase (EC 2.7.1.1) (4 in Figure 1). Fructose-6-phosphate (F-6-P) is converted into glucose-6-phosphate (G-6-P) by glucose phosphate isomerase (EC 5.3.1.9) (5 in Figure 1), but on the other hand, F-6-P can synthesis sucrose via sucrose phosphate synthase (SPS, EC 2.4.1.14) (6 in Figure 1). Fourthly, sucrose is reversibly converted into fructose and uridine diphosphate glucose (UDPG) by sucrose synthase (SuSy, EC 2.4.1.13) (7 in Figure 1). Then UDPG is further metabolized to glucose-1-phosphate (G-1-P) by the action of UDPG pyrophosphorylase (UGPase, EC 2.7.7.9) (8 in Figure 1). G-1-P, which can also be transformed from G-6-P by phosphoglucomutase (PGM, EC 2.7.5.1) (9 in Figure 1), serves as a precursor of adenosine diphosphate glucose (ADPG) by ADPG pyrophosphorylase (AGPase, EC 2.7.7.27) (10 in Figure 1) [16]. Both G-1-P and G-6-P are translocated into the amyloplasts via phosphate translators (11 in Figure 1), whereas ADPG is translocated via ADPG transporters (12 in Figure 1). Then, starch biosynthesis occurs in the amyloplast. Starch can be chemically classified into two homopolymers: amylose and amylopectin. Amylose is an almost linear α-1,4 glucan molecule synthesized by AGPase and granule-bound starch synthase (GBSS, EC 2.4.1.21) whereas amylopectin is a highly branched glucan achieved by a coordinated series of enzymatic reactions involving AGPase, soluble starch synthase (SSS, EC 2.4.1.21), starch branching enzyme (SBE) (EC 2.4.1.18), and starch de-branching enzyme (SDBE, EC 3.2.1.10). The rate-limiting step is the synthesis of ADPG from G-1-P and ATP by AGPase [17]. Following the ADPG catalytic reaction, SSS catalyses the transfer of a glucosyl unit from ADPG to the reducing end of the glucose chain [18] (13 in Figure 1). After elongation of the glucan chains by SSS, SBE generates amylopectin by cleaving α, 1–4 glucosidic bonds and transferring the released reducing end to C6 hydroxyls to form an α, 1–6 branch point. Following starch branching, SDBE catalyzes the hydrolysis of α, 1–4 bonds [19]. In particular, GBSS is mainly responsible for the synthesis of amylose and long amylopectin chains (14 in Figure 1).

To date, some research on starch or sucrose metabolism has been reported in some bulbous plants, including *Gladiolus hybridus* [20] and *Tulipa gesneriana* [21]. These studies indicate that starch and sucrose metabolism is crucial for the formation and development of bulblets. Similar to other bulbs, the development of lily bulbs can be achieved by using photoassimilates for morphogenesis and the accumulation of reserve metabolites. Sun et al. [22] found that sucrose was the predominant transported form of photoassimilates in the phloem of *L. davidii* var. *unicolor*, from photosynthetic leaves to bulbs, where it accounted for most (~70%) of total soluble sugar; moreover, during scale cutting propagation, starch content in mother scales declined while a synchronous increase occurred in bulblets [9]. Starch was hydrolyzed in the bulb scales and sugars accumulated resulting in the increase of reproductive capacity during storage at a low temperature (5°C), which indicated that the preparation for later bulb growth involves mobilization of carbohydrate reserves [23,24]. These clues from the literature indicate that sucrose and starch metabolism have a key function in bulblet formation and development. Despite the significance of carbohydrates in the formation and development of lily bulblets, information on the role of carbohydrate metabolism and related genes is very limited. Few studies on the variation of carbohydrate compounds during bulblet development have been reported yet [24,25]. For enzymes involved in sucrose and starch catabolism, low temperature (4°C) resulted in increasingα-amylase, β-amylase, SPS and SuSy (in the synthesis direction) activity, but decreasing invertase (INV) activity in bulblets regenerated *in vitro* [26]. Castro and Clément [27] suggested that CWIN might be essential for soluble sugar partitioning in different fractions of the anther. However, the molecular mechanism underlying carbohydrate metabolism regulating bulblet development in *Lilium* is still enigmatic. The classification of gene expression patterns associated with specific stages of bulblet formation and development and functional characterization of the encoded genes are critical aspects for understanding the molecular and biochemical events associated with bulblet development.

To broaden our knowledge of *Lilium* global gene expression profiles and to identify the genes involved in bulblet formation and development, this is the first study to compare gene expression profiles during bulblet formation and development in the *Lilium* genus by taking advantage of the next-generation high-throughput sequencing platforms Illumina GAIIx and HiSeq 2000 to sequence the transcriptome of *L. davidii* var. *unicolor* bulblets. Furthermore, the analysis presented in this study identifies most carbohydrate genes and biological functions regulating bulblet development for the first time and highlights important biological processes associated with bulblet development in *Lilium*.

## Results

### Collection of bulblets

**Figure 2:**
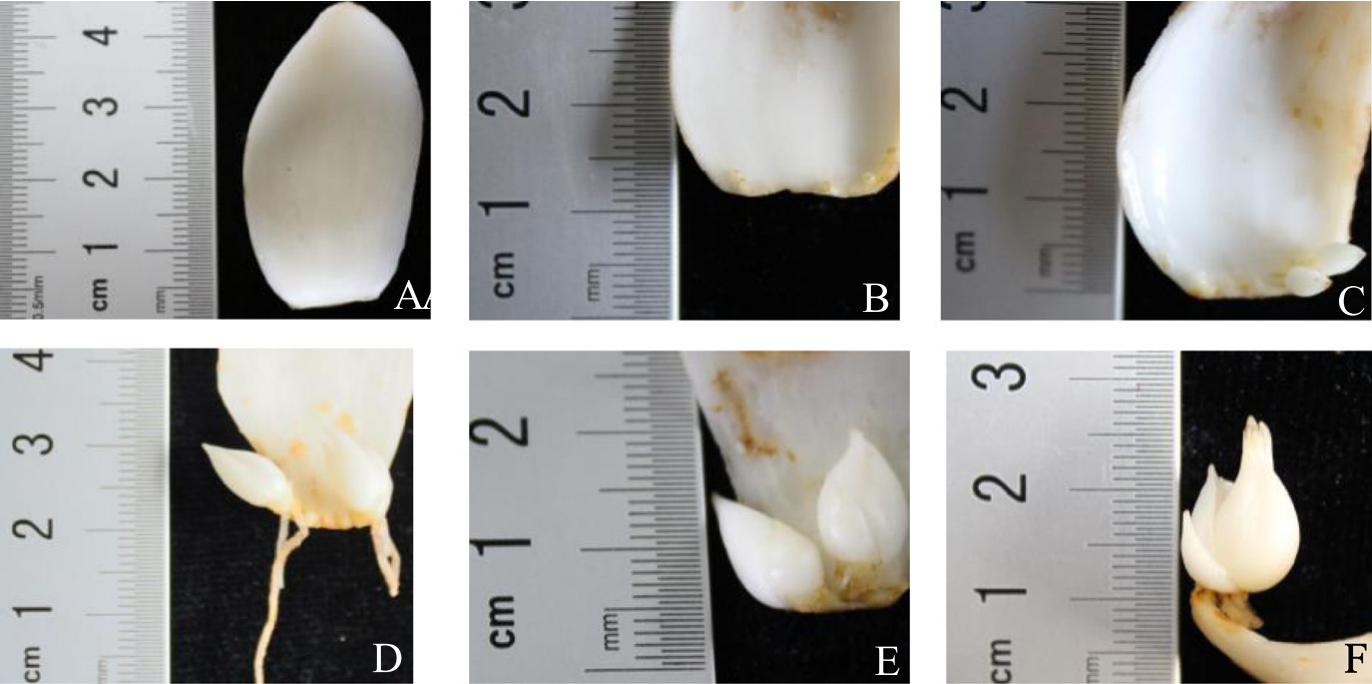
**Developing stages during bulbing in** ***L. davidii*** **var** ***. unicolor*** **. A** Stage of scale removal from the parent bulb (0 d). **B** Stage of bulblet-prototype appeared at base of mother scales (2 weeks after scale cutting). **C** Stage of bumps that developed into bulblets with a basic shape (35 d after scale cutting). **D** Stage of bulblets with complete shape (42 d after scale cutting). **E** and **F** Stages of bulblet expansion (56 d and 70 d after scale cutting).

The transcriptome of *L. davidii* var. *unicolor* bulblets during development was assessed, including physiological and morphological changes during a three-phase process. The first phase is the appearance of bulblets, about 2 weeks after incubation at 25°C. Rudimentary bulblets, represented by 1–3 small white bumps, emerged at the basal end of the adaxial side of mother scales (Figure 2B). The second stage is bulblet formation, represented by bumps that developed into bulblets with a basic shape, a further 2 weeks later (Figure 2C). The third phase of bulblet development, which involved considerable enlargement and growth (Figure 2D-F), saw a distinct and pronounced change in bulblet size.

### Sequencing and assembly

After strict quality control, 10–17 million (M) reads with ~95% Q20 bases were selected as high quality reads, and were used in subsequent analysis (Table 1). Using Trinity, the reads were assembled into 52,901 unigenes with a mean length of 630 bp. The N50 contig was 926 bp long and 10,745 unigenes had longer sequences. On average, 70% of these uniquely mapped to the reference genome (Table 2). 52,652 coding DNA sequences (CDSs) were predicted to have a mean length of 526 bp.

**Table 1:** **Description of three** ***L. davidii*** **var.** ***unicolor*** **RNA-seq libraries**

**Stage**	**Cycle number**	**Total reads**	**Total bases**	**GC (%)**	**Q20 (%)**
0 d	100	11840388	3525591648	50.5	86.87
15 d	100	10441093	2391758376	50.2	87.52
35 d	100	17453424	1999999993	49.3	89.91

**Table 2:** **Summary of alignment of** ***L. davidii*** **var.** ***unicolor*** **RNA-seq libraries**

**Stage**	**Total reads**	**Contigs**	**Mapped reads (%, mapped/total)**	**Perfect mapped reads (%, perfect/mapped)**	**Uniquely mapped reads (%, unique/mapped)**
0 d	11840388	474906	8353030 (70.5)	7293360 (87.31)	8226995 (98.49)
15 d	10441093	1014316	6999543 (67.0)	5198591 (74.27)	6847117 (97.82)
35 d	17453424	651981	12271124 (70.3)	10761028 (87.69)	12098504 (98.59)

### Gene annotation and function classification

**Figure 3:**
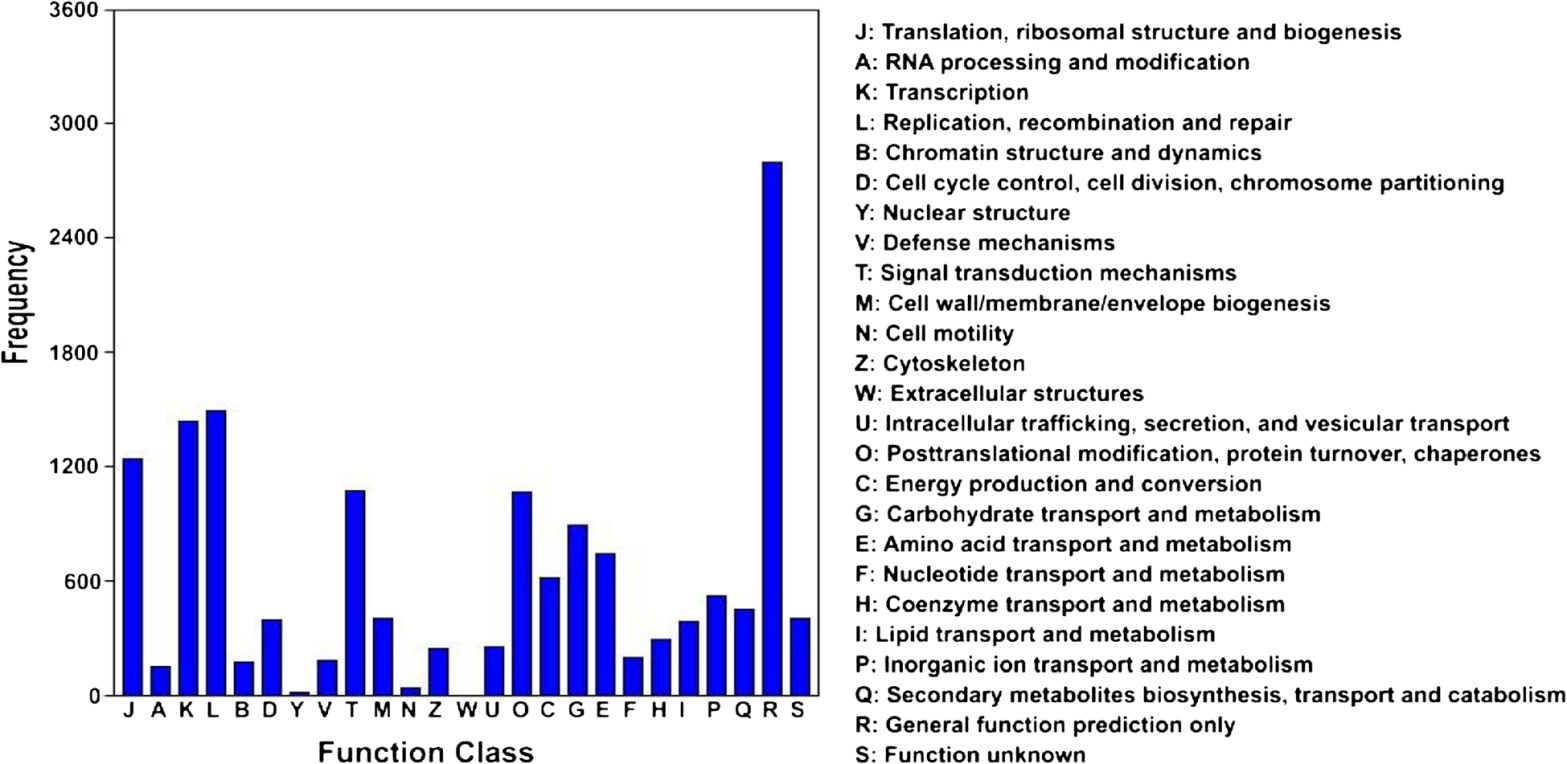
**COG function classifications in** ***L. davidii*** **var.** ***unicolor.***

The assembled unigenes were searched against Swiss-Prot and TrEMBL protein databases to achieve validation and annotation, resulting in 37,385 (70%) annotated unigenes. Among them, 27,098 (52.02% of the total) and 35,241 (66.62% of the total) had significant similarity to known proteins in Swiss-Prot and TrEMBL protein databases, respectively. To further evaluate the completeness of the transcriptome, we randomly searched the annotated sequences for genes with COG classifications. As a result, 11,150 sequences were classified into 24 COG categories (Figure 3). At the very top, the clusters were general function, replication, transcription, and translation. In addition, carbohydrate transport and metabolism held a central position with 891 unigenes (i.e., 8% of annotated COG).

There were 26,333 unigenes that could be successfully annotated according by BLAST2GO (Additional file 1: Figure S1). The main distributions in the molecular category were catalytic activity (13,128), binding (12,572), transporter activity (1,738), structural molecule activity (794), and nucleic acid binding transcription factor activity (633). In the cell component, most frequent distributions were in the cell (18,672), cell parts (18,666), organelles (15,227), membranes (7,977), and organelle parts (4,982). The most representative distributions in the biological process category were as follows: metabolic processes (17,756), cellular processes (16,699), responses to stimuli (8,332), biological regulation (7,006), and cellular component organization or biogenesis (5,153). Among all the functions, there were many processes involved in carbohydrate metabolism (Additional file 2: Table S1).

### Gene expression during bulblet development

The level of gene expression was determined by calculating the number of reads for each gene and then normalizing this to RPKM. Most unigenes were expressed at low levels whereas a small proportion of unigenes were highly expressed (Additional file 3: Figure S2). The variations in gene expression during bulblet development were analyzed using the IDEG6 program to identify two-fold up-regulated and down-regulated genes with a *P* value < 0.01. Finally, 3,337 differentially expressed genes (DEGs) were identified. As shown in Additional file 4: Figure S3, between 35 and 0 d, the gene-expression pattern changed steadily, but not dramatically (695 up-regulated vs 668 down-regulated genes). Between 35 and 15 d, 1,964 DEGs were detected with 675 up-regulated and 1,289 down-regulated genes. The greatest changes in gene expression occurred at 15 d compared with 0 d. Between 15 and 0 d, a total of 2,421 up-regulated and 652 down-regulated genes were detected.

### Pathway enrichment analysis of DEGs

KEGG pathway enrichment analysis was performed to categorize the biological functions of DEGs. We mapped all the genes to terms in the KEGG database. Specific enrichment of genes was observed for 220 pathways in the 0 d vs 15 d comparison, 198 pathways in the 15 d vs 35 d comparison, and 170 pathways in the 0 d vs 35 d comparison. The first 15 enriched pathways between library pairs are listed in Additional file 5: Table S2. Notably, starch and sucrose metabolism constituted the primary pathway among the three library pairs. The expression of starch and sucrose metabolism genes is shown in Additional file 6: Table S3. The other pathways were mainly involved in secondary metabolism, energy metabolism, splicing, and protein synthesis. These results agree with the findings from the DEG analysis and suggest that starch and sucrose metabolism pathways might be more active during bulblet formation and development.

### Starch and sucrose concentration

**Figure 4:**
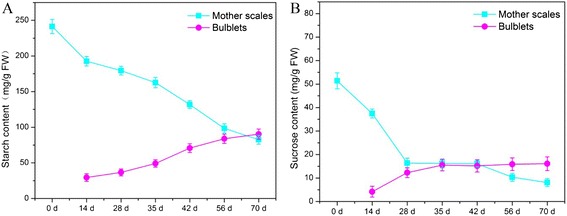
**Changes of content of starch and sucrose in mother scales and bulblets of** ***L. davidii*** **var.** ***unicolor.*** **A**: starch content in mother scales and bulblets **B**: sucrose content in mother scales and bulblets.

Breakdown of carbohydrate reserves was measured in mother scales from 0 d to 70 d (Figure 4). In the first 14 d, starch strongly decreased in mother scales to provide energy for the activation of bulblet formation; starch then continued to decline slowly (Figure 4A). From 35 d onwards, starch content in mother scales once again fell sharply, suggesting that the development and enlargement of bulblets need nutrients and energy supplied by starch hydrolysis. Meanwhile, the starch content in bulblets increased gradually during the entire development process, especially at the late developmental stage (35–70 d).

Sucrose content changed mainly at the beginning of bulblet formation (Figure 4B), decreasing sharply in mother scales but increasing equally sharply in bulblets. Hereafter, although the speed of starch degradation accelerated, the content of sucrose in mother scales and bulblets showed a steady decrease and increase, respectively.

### Verification of gene expression by quantitative real-time PCR

In order to verify the gene expression profiles of enzymes involved in starch and sucrose metabolism obtained from the RNA-seq approach, quantitative real-time PCR was utilized to analyze the expression of 16 selected genes, which encoded AGPase (*AGP1*, *AGP2*, and *AGP3*), INV (*INV*1 and *INV2*), SuSy (*SuSy1*, *SuSy2*, and *SuSy3*), SPS (*SPS1* and *SPS2*), SBE (*SBE*), SDBE (*SDBE1* and *SDBE2*), GBSS (*GBSS*), and SSS (*SSS1* and *SSS2*). The results of agarose gel electrophoresis demonstrated that all 16 primer pairs amplified a single band of expected size (Additional file 7: Figure S4). The correlation coefficients (R^2^) ranged in value between 0.9900 and 1.0000, and PCR amplification efficiencies between 94 and 105% were obtained from the standard curves generated using a 10-fold serial dilution of cDNA (Additional file 8: Table S4). Based on the RPKM in different libraries, *GAPDH* was selected as the reference gene due to its almost stable expression (|log2Ratio| ≤ 0.5), with a |log2 (0 d/15 d)| value of 0.4494, a |log2 (0 d/35 d)| value of 0.2227, and a |log2 (35 d/15 d)| value of 0.2267.

**Figure 5:**
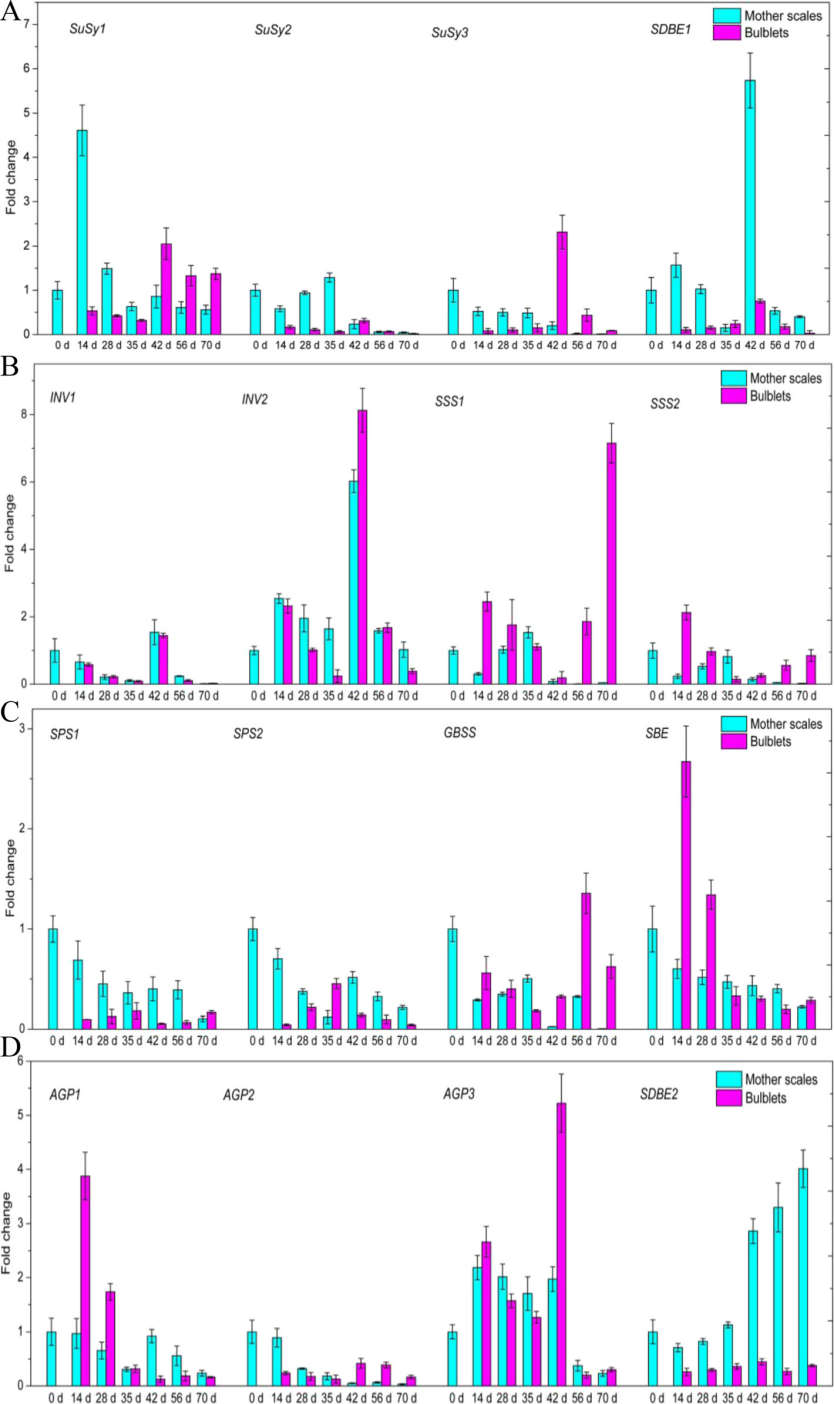
**Expression profiles of 16 genes in mother scales and bulblets of** ***L. davidii*** **var.** ***unicolor*** **by the quantitative real-time PCR.** Values for quantitative real-time PCR are means ± SE of three replicates. **A**: Expression profiles of *SuSy1*, *SuSy2*, *SuSy3* and *SDBE1*; **B**: Expression profiles of *INV1*, *INV2*, *SSS1* and *SSS2*; **C**: Expression profiles of *SPS1*, *SPS2*, *GBSS* and *SBE*; **D**: Expression profiles of *AGP1*, *AGP2*, *AGP3* and *SDBE2*.

The expression pattern analyzed by quantitative real-time PCR was almost consistent with that observed in transcriptome analysis (Additional file 6: Table S3). At bulblet appearance (14 d) and morphogenesis (28–35 d), the expression of SuSy homologous genes in mother scales was higher than in bulblets, while during bulblet enlargement (42–70 d), the expression of SuSy genes in bulblets was higher (Figure 5A). Both in mother scales and bulblets, the dominant SuSy gene was *SuSy1*. However, *SuSy3* showed a more drastic change – nearly 30 fold – in bulblets than the other two SuSy genes. Meanwhile, *INV2* presented a relatively high expression level and dramatic shift in mother scales and bulblets than *INV1* (Figure 5B). SPS genes, in contrast, showed relatively low and stable expression both in mother scales and bulblets (Figure 5C). In mother scales, the expression of SPS homologous genes gradually decreased during bulblet formation and enlargement, and increased slightly at 42 d. In bulblets, both *SPS1* and *SPS2* peaked at 35 d, which corresponds to bulblet formation. Compared with *SPS1*, *SPS2* changed more sharply. For enzymes involved in starch metabolism, those in the synthetic direction such as AGPase, SSS, SBE and GBSS, showed a decreasing trend in mother scales and higher gene expression at bulblet appearance and enlargement stages (Figure 5B,C, and D), while enzymes in the cleavage direction (SDBE) showed higher gene expression in mother scales than in bulblets (Figure 5A and D).

## Discussion

### *Lilium* bulblet formation and development transcriptome

High throughput transcriptome sequencing by next generation sequencing platforms Illumina GAIIx and HiSeq 2000 is a powerful and efficient approach for gene expression analysis at the genome level. To date, the RNA-seq approach is widely used to investigate the transcriptome of plants for which the whole genome is known but is especially suitable for gene expression profiling in non-model organisms that lack knowledge of genomic sequences.

Despite the economic importance of *Lilium*, its genome is not yet publically available and there is limited sequence data [28-30]. Besides, there are vast differences in cell ploidy and genetic characteristics of different *Lilium* genotypes. As far as the authors are aware, this is the only report to use RNA-seq to identify large number of genes involved in lily bulblet formation and development. In this study of *L. davidii* var. *unicolor* (2n = 24), 52,901 unigenes were obtained, demonstrating the successful use of the RNA-seq approach to profile gene expression in a species without a fully sequenced genome. Among them, 37,385 unigenes were successfully annotated but about 30% of the genes in our transcriptome could not have functions assigned as a result of the limitation of genome information in *Lilium*.

To obtain all the DEGs from RNA-seq data, the expression of all genes was analyzed depending on the RPKM. Based on the comparative analyses of the RNA-seq datasets and public information about the metabolic pathway, one objective was to narrow down the number of candidate genes responsible for bulblet formation and development. Finally, a large number of DEGs corresponding to starch and sucrose metabolism were detected, in addition to secondary metabolism, energy metabolism, splicing, and protein synthesis. These results indicate that the morphogenesis and growth of bulblets demanded the participation of carbohydrates besides genetic material. At 15 d, bulblets formed from scratch, therefore many of the genes associated with the early events of bulblet emergence were highly expressed. Correspondingly, the greatest changes in gene expression occurred at 15 d compared with 0 d, with a total of 2,421 up-regulated and 652 down-regulated genes. These temporal gene expression patterns indicate that the stage of bulblet appearance (15 d) was the most active stage. Distributing transcripts in GO categories provided a molecular snap shot of bulblet formation and development. The biological process was the most prominent GO category (52%), and about 24% of all transcripts fell under the molecular function. Among all functions, there were many processes involved in carbohydrate metabolism, suggesting that genes involved in carbohydrate metabolism play an important role in bulblet formation and development. The bulblet is an energy sink tissue for plant reproduction in which starch and sucrose are mobilized for photosynthetic organs and are broken down to sugars, which function as a precursor to essential metabolites. According to COG classifications, carbohydrate transport and metabolism held a central position, indicating that the morphogenesis and growth of bulblets demanded the participation of carbohydrates. This was also the viewpoint supported by the transcript abundance of different KEGG pathways.

### Starch and sucrose metabolism in different plants

Starch and sucrose metabolism is vital to plant development and the response to abiotic stress. Firstly, starch and sucrose metabolism is of great significance in seed development. Many DEGs related to starch/sugar metabolism were found in seeds of rice (*Oryza sativa* L.) *sugary* mutant, more than in the wild type ‘Sindongjin’. Detailed pathway dissection and quantitative real time PCR demonstrated that most genes involved in sucrose to starch synthesis were up-regulated, whereas the expression of the AGPase small subunit was specifically inhibited during the grain-filling stage in the *sugary* mutant [31]. In wheat (*Triticum aestivum* L. cv. ‘Butte 86’) grain, high temperature restrained the accumulation of starch with the concomitant lower expression of AGPase, SSS, GBSS, and SBE genes [32].

Starch and sucrose metabolism is also crucial to fruit development. Gene set enrichment analysis suggested that glycolysis and carbohydrate metabolism were significantly altered in puffed *Citrus* fruit, with higher gene expression of INV and SBE, as well as lower gene expression of AGPase and SSS [33]. Meanwhile, starch and sucrose metabolism is high during the development and ripening of mango (*Mangifera indica* Linn.) fruits [34]. During pineapple (*Ananas comosus*) fruit development, the large rise in sucrose was accompanied by dramatic up-regulated changes in SPS, and a cycle of sucrose breakdown in the cytosol of sink tissues could be mediated both by SuSy and INV [35].

Starch and sucrose metabolism are dominant during flower blooming in *Rosa chinensis* ‘Pallida’ [36] and are also responsible for the increase of cold tolerance in blueberry (*Vaccinium* spp.) [37] as well as *Lilium lancifolium* [29], due to the accumulation of soluble sugars. Moreover, starch and sucrose was responsible for subterranean organ formation such as in sweetpotato (*Ipomoea batatas* L.) [38] and potato (*Solanum tuberosum* L.) [39]. The present study of the *L. davidii* var. *unicolor* transcriptome revealed a large number of genes involved in carbohydrate metabolism (Additional file 2: Table S1). According to KEGG pathway analysis, starch and sucrose metabolism constituted the primary pathway in the three RNA-seq libraries (Additional file 5: Table S2). All these facts support the fact that carbohydrates are vital to bulb formation and development of bulbous ornamentals [8,9].

### Gene expression during bulblets development

SuSy can catalyze sucrose catabolism reversibly but INV catalyzes irreversibly. However, SuSy is generally considered to be involved in sucrose hydrolysis rather than sucrose synthesis [10]. SPS is crucial to carbohydrate metabolism by regulating carbon partitioning between starch production and carbohydrate accumulation. In our study, SuSy and INV genes showed relatively higher expression, but SPS genes showed an overall decline in mother scales (Figure 5A and C), in accordance with the progressive decrease of sucrose content in mother scales (Figure 4B). This result indicates that sucrose in mother scales is mainly metabolized in the cleavage direction to provide energy for bulblet formation and development. In spite of their relatively faint expression, both SPS homologous genes demonstrated high activity in bulblets during bulblet formation (28–35 d), in accordance with the bare expression of SuSy and INV family genes, which indicates that sucrose plays a critically important role in bulblet morphogenesis. This is because sucrose serves as a critical signaling molecule in relation to cellular metabolic status [40], and as a signal in the regulated expression of microRNAs, which are transcription factors that modulate plant development [12]. When bulblets began to swell at 42 d, the expression of SuSy and INV peaked quickly, causing the hydrolysis of sucrose to provide carbon skeletons for starch synthesis. The expression pattern of SuSy and INV at different phases suggests that SuSy and INV work cooperatively to cleave sucrose.

An increase in amylose content can be accomplished by inhibiting enzymes involved in amylopectin synthesis [41,42], by raising the expression levels of *GBSSI* [43], or simultaneously by raising the expression levels of *AGP* as well as *GBSS* and suppressing the expression levels of *SBE* [18]. However, amylopectin is the main form of starch in lily, accounting for 70% of all starch [44]. This was demonstrated by the gene expression of enzymes involved in amylopectin synthesis, namely SSS, SBE, and AGPase (Figure 5B,C,D), which was considerably higher than the expression of the amylose synthesis enzyme *GBSS* (Figure 5D). Another piece of evidence was that SDBE homologous genes were highly expressed in mother scales. This is because both SDBE homologous genes found in *L. davidii* var*. unicolor* fall into the PUL group, which has been proposed to participate in amylopectin disassembly [45]. The expression levels of genes involved in starch synthesis (i.e., AGPase, SBE, SSS, and GBSS) in bulblets were distinctly higher than those in mother scales (Figure 5B,C,D), while genes involved in starch hydrolysis demonstrated an opposite trend. The change in starch metabolism-related gene expression was in accordance with the starch content in mother scales and bulblets (Figure 4A). In bulblets, genes involved in starch synthesis were most expressed at 14 d and later stages (35–70 d) of bulblet development, but rarely expressed at 35 d (Figure 5C,D), indicating that starch is pivotal to bulblet emergence and development.

## Conclusions

In the present study, using three developmental stages of bulblets of *Lilium davidii* var. *unicolor*, which does not have a reference genome, we performed comparative gene expression at the transcriptional scale by using RNA-seq. The transcriptome was assembled with Trinity and functionally annotated with Blast2GO and KEGG. A set of genes that might contribute to starch and sucrose metabolism were identified and genetic mechanisms of these genes related to bulblet development were discussed. Gene expression, together with changes in the content of starch and sucrose, suggest that sucrose is crucial for bulblet morphogenesis while starch is vital to bulblet emergence and development. The results will certainly be valuable for elucidation of molecular mechanisms in bulblet emergence and development in *Lilium* and related species.

## Methods

### Plant material and processing

Fresh *L. davidii* var. *unicolor* bulbs (12–14 cm in circumference) were obtained from the Gansu Academy of Agricultural Sciences, Lanzhou (103.4°E, 36°N), PR China. The ability to produce bulblets decreases from external to internal scales [46]. Thus, healthy external scales without damage caused by disease or pets were removed carefully from the mother bulbs, washed in running water to remove dirt, surface sterilized by immersing in 0.01% potassium permanganate solution for 20 min, and then washed with distilled water three times using an in-house protocol. After surface sterilization, scales (three biological replicates, 150 scales in each) were embedded concave upward *ex vitro* into pre-sterilized (180°C for 5 h) wet peat substrate (XinYuan Gardening Resources Ltd., Liaoning, PR China) of 60% relative humidity, with 90 scales/300 cm^2^ (60 cm × 5 cm). Propagules were placed into perforated plastic bags (60 cm × 90 cm) and then incubated at 25°C in the dark.

### Sample collection

The propagation of bulblets was investigated 70 days after embedding in peat when the bulblets formed a definite shape (bulbous) and size (2 cm circumference with 5–7 scales). To construct the cDNA library, samples were collected at 0 and 15 d (appearance of bulblets), and 35 d (bulblets formed a basic shape with 3–4 scales). For quantitative real-time PCR, mother scales and bulblets were randomly collected at 0 d, 14 d, 28 d, 35 d, 42 d, 56 d, and 70 d. Using 15 samples from triplicate treatments constituted three biological replicates for experiments. Samples were flash frozen in liquid nitrogen and stored at −80°C until use.

### RNA extraction, cDNA library construction, sequencing and assembly

Total RNA was extracted from frozen mother scales and bulblets separately according to Li et al. [47]. The purity and concentration of total RNA were determined using an Infinite® 200 PRO (Tecan, Männedorf, Switzerland) as well as RNA gel electrophoresis (formaldehyde buffer system). The cDNA libraries of 0 d, 15 d and 35 d were constructed using the mRNA Sequencing Sample Preparation Kit following the manufacturer’s (Illumina, CA, USA) instructions. cDNA fragments 200 ± 25 bp in size were selected for PCR amplification. Finally, sequencing was performed on the Illumina cluster station and Illumina Genome Analyzer IIx sequencing platform. The clean reads generated were then used for all subsequent analyses. Trinity (http://trinityrnaseq.sourceforge.net/) was used to assemble the pair-end short reads into contigs.

### Gene functional annotation

The read sequences were searched against NCBI Nr and Nt, Swiss-Prot, and TrEMBL databases using BLAST with an e-value of 10^−5^. Gene names were assigned to each assembled sequence based on the best hit (highest score). Open reading frames (ORFs) were predicted using the ‘getorf’ program of EMBOSS software package. The Blast2GO program was used to analyze Gene Ontology annotation (GO, http://www.geneontology.org). The sequences were also aligned to the Clusters of Orthologous Groups (COG) database (http://www.ncbi.nlm.nih.gov/COG/) to predict and classify functions. The Kyoto Encyclopedia of Genes and Genomes (KEGG) pathways were assigned to the sequences using the online KEGG Automatic Annotation Server (KAAS) (http://www.genome.jp/kegg/kaas/). All these searches were performed with a cut-off e-value of 10^−5^.

### Differential gene expression analyses

To compare gene expression abundance in different samples, transcript count information for sequences corresponding to each unigene was calculated and normalized to the reads per kilobase of exon model per million mapped reads (RPKM) values [48]. Significant DEGs were determined by using a general chi-squared test integrated in IDEG6 software (http://telethon.bio.unipd.it/bioinfo/IDEG6/) [49]. P values from this method were adjusted to account for multiple tests using the false discovery rate (FDR). Genes with an adjusted P value < 0.01 and an absolute value of log2 (expression fold change) ≥1 were deemed to be differentially expressed. Fold changes of expression levels between samples were calculated.

### Starch and sucrose assays

The carbohydrates in mother scales and bulblets were determined. Starch content was determined by iodine colorimetry while sucrose was separated by HPLC [9]. The content of carbohydrates in mother scales and bulblets was determined in three independent experiments. Both experiments were carried out with three independent biological replicates.

### Quantitative real-time RT-PCR

To verify RNA-seq results and to determine the roles of key enzymes involved in sucrose and starch metabolism, quantitative real-time PCR was conducted using SYBR® green (CWBIO, Beijing, China) and an ABI 7500 Real-Time PCR System (Life Technologies, CA, USA). First-strand cDNA was synthesized from 2 μg of DNase I-treated total RNA using M-MLV Reverse Transcriptase (Promega, Madison, USA). SYBR® green primers for quantitative real-time RT-PCR were designed using Primer Premier 5.0 software with melting temperatures (*Tm*) of 55-65°C, primer length of 17–25 bp, and amplicon length between 85 and 300 bp (Additional file 8: Table S4). To ensure target specificity, gene sequences were blasted against the NCBI database to determine cross homology with other sequences. The primer specificities were confirmed on 2% agarose gel electrophoresis for a single product giving the expected size. Quantitative real-time PCR was carried out in a total volume of 20 μl containing 0.8 μl of template, 0.2 μM of each primer combination, and 1× UltraSYBR Mixture (with ROX). The following amplification program was used: denaturation at 95°C for 10 min, 44 cycles of amplification (95°C for 30 s, 60°C for 30 s, 68°C for 1 min) and a melting curve program (95°C for 15 s, 60°C for 1 min, 95°C for 30 s, 60°C for 15 s). All PCR reactions were performed in biological triplicates on 96-well PCR plates (Corning, NY, USA). Relative mRNA levels were calculated using the 2^-ΔΔCt^ method against the internal control glyceraldehyde-3-phosphate dehydrogenase (GAPDH). To estimate PCR efficiencies, standard curves of a 10-fold dilution series from pooled cDNA was made to calculate the gene-specific PCR efficiency and regression coefficient (R^2^) for each gene.

### Availability of supporting data

The sequence datasets supporting the genes used in this article are available at NCBI from accession number KP179405 to KP179417.

## Additional files

Additional file 1: Figure S1. Functional classification of the unigenes derived from *L. davidii* var. *unicolor* according to Gene Ontology classifications including attributions of **(1)** molecular function, **(2)** cellular component, and **(3)** biological process. Additional file 2: Table S1. Numbers of unigenes involved in carbohydrate metabolism. Additional file 3: Figure S2. Distribution of gene expression levels. Additional file 4: Figure S3. Changes in gene expression profiles among different stages. Additional file 5: Table S2. List of first 15 pathways between library pairs. Additional file 6: Table S3. Expression of starch-sucrose metabolism genes of *L. davidii* var. *unicolor*. Additional file 7: Figure S4. Gene specificity and amplicon size. Agarose gel (2%) electrophoresis showing amplification of a specific PCR product of expected size for each gene. M presents 2,000 bp DNA marker. Additional file 8: Table S4. Primers used in quantitative real-time PCR of *L. davidii* var. *unicolor*.

## Abbreviations

ADPG: Adenosine diphosphate glucose
AGPase: ADPG pyrophosphorylase
CDSs: Coding DNA sequences
CIN: Cytoplasmic invertase
COG: Clusters of Orthologous Groups
CWIN: Cell wall invertase
F-6-P: Fructose-6-phosphate
G-1-P: Glucose-1-phosphate
G-6-P: Glucose-6-phosphate
GBSS: Granule-bound starch synthase
GAPDH: Glyceraldehyde-3-phosphate dehydrogenase
GO: Gene Ontology
INV: Invertase
KEGG: Kyoto Encyclopedia of Genes and Genomes
ORF: Open reading frame
PGM: Phosphoglucomutase
RPKM: Reads per kilobase of exon model per million mapped reads
SBE: Starch branching enzyme
SDBE: Starch de-branching enzyme
SPS: Sucrose phosphate synthase
SSS: Soluble starch synthase
SuSy: Sucrose synthase
UDPG: Uridine diphosphate glucose
UGP: UDPG pyrophosphorylase
VIN: Vacuolar invertase
