# Supplementary material for: Transcriptome analysis of carbohydrate metabolism during bulblet formation and development in Lilium davidii var. unicolor
Source: BMC Plant Biol. 2014 Dec 19;14:358. doi: 10.1186/s12870-014-0358-4 (PMC4302423; doi:10.1186/s12870-014-0358-4)
Supplement: Additional file 1: Figure S1. — Functional classification of the unigenes derived from L. davidii var. unicolor according to Gene Ontology classifications including attributions of (1) molecular function, (2) cellular component, and (3) biological process. [file 12870_2014_358_MOESM1_ESM.doc]

(a)

(b)

(c)

(d)

Magnified view

(a)

(b)

(c)

(d)

(e)

(f)

Magnified view

(e)

(f)

(g)

Magnified view

(g)

(h)

(h)

(2)

(3)

(1)
