# Supplementary material for: Transcriptome analysis of carbohydrate metabolism during bulblet formation and development in Lilium davidii var. unicolor
Source: BMC Plant Biol. 2014 Dec 19;14:358. doi: 10.1186/s12870-014-0358-4 (PMC4302423; doi:10.1186/s12870-014-0358-4)
Supplement: Additional file 2: Table S1. — Numbers of unigenes involved in carbohydrate metabolism. [file 12870_2014_358_MOESM2_ESM.doc]

| **Table S1**: Numbers of unigenes involved in carbohydrate metabolism | | | |
| --- | --- | --- | --- |
| Function | Unigene number | GO-ID | GO category |
| carbohydrate metabolic process | 326 | GO:0005975 | Biological Process |
| glycolysis | 274 | GO:0006096 | Biological Process |
| starch biosynthetic process | 272 | GO:0019252 | Biological Process |
| gluconeogenesis | 200 | GO:0006094 | Biological Process |
| glucose catabolic process | 123 | GO:0006007 | Biological Process |
| polysaccharide biosynthetic process | 121 | GO:0000271 | Biological Process |
| carbohydrate binding | 119 | GO:0030246 | Molecular Function |
| carbohydrate transport | 66 | GO:0008643 | Biological Process |
| starch metabolic process | 56 | GO:0005982 | Biological Process |
| starch catabolic process | 41 | GO:0005983 | Biological Process |
| carbohydrate biosynthetic process | 38 | GO:0016051 | Biological Process |
| cellular carbohydrate metabolic process | 36 | GO:0044262 | Biological Process |
| beta-amylase activity | 30 | GO:0016161 | Molecular Function |
| sucrose metabolic process | 26 | GO:0005985 | Biological Process |
| galactose metabolic process | 25 | GO:0006012 | Biological Process |
| polysaccharide binding | 24 | GO:0030247 | Molecular Function |
| sucrose synthase activity | 17 | GO:0016157 | Molecular Function |
| sucrose biosynthetic process | 16 | GO:0005986 | Biological Process |
| regulation of carbohydrate metabolic process | 15 | GO:0006109 | Biological Process |
| sucrose alpha-glucosidase activity | 14 | GO:0004575 | Molecular Function |
| galactinol-sucrose galactosyltransferase activity | 14 | GO:0047274 | Molecular Function |
| sucrose:hydrogen symporter activity | 13 | GO:0008506 | Molecular Function |
| diphosphate-fructose-6-phosphate 1-phosphotransferase activity | 13 | GO:0047334 | Molecular Function |
| sucrose-phosphate synthase activity | 11 | GO:0046524 | Molecular Function |
| starch binding | 10 | GO:2001070 | Molecular Function |
| sucrose transport | 10 | GO:0015770 | Biological Process |
| glucose 6-phosphate metabolic process | 10 | GO:0051156 | Biological Process |
| dTDP-glucose 4,6-dehydratase activity | 9 | GO:0008460 | Molecular Function |
| glucose metabolic process | 8 | GO:0006006 | Biological Process |
| starch synthase activity | 7 | GO:0009011 | Molecular Function |
| sucrose catabolic process | 7 | GO:0005987 | Biological Process |
| UDP-glucose transmembrane transporter activity | 7 | GO:0005460 | Molecular Function |
| carbohydrate phosphorylation | 7 | GO:0046835 | Biological Process |
| UDP-glucose transport | 6 | GO:0015786 | Biological Process |
| UDP-glucose 4,6-dehydratase activity | 6 | GO:0050377 | Molecular Function |
| UDP-glucose 6-dehydrogenase activity | 6 | GO:0003979 | Molecular Function |
| regulation of carbohydrate biosynthetic process | 5 | GO:0043255 | Biological Process |
| UDP-glucose metabolic process | 4 | GO:0006011 | Biological Process |
| glucose-6-phosphate transport | 4 | GO:0015760 | Biological Process |
| UTP:glucose-1-phosphate uridylyltransferase activity | 4 | GO:0003983 | Molecular Function |
| fructose-2,6-bisphosphate 2-phosphatase activity | 4 | GO:0004331 | Molecular Function |
| fructose metabolic process | 4 | GO:0006000 | Biological Process |
| carbohydrate homeostasis | 4 | GO:0033500 | Biological Process |
| carbohydrate derivative metabolic process | 3 | GO:1901135 | Biological Process |
| amyloplast starch grain | 2 | GO:0009568 | Cellular Component |
| ADP-glucose pyrophosphohydrolase activity | 2 | GO:0080042 | Molecular Function |
| glucose-6-phosphate isomerase activity | 2 | GO:0004347 | Molecular Function |
| fructose 6-phosphate metabolic process | 2 | GO:0006002 | Biological Process |
| fructose 2,6-bisphosphate metabolic process | 2 | GO:0006003 | Biological Process |
| carbohydrate catabolic process | 2 | GO:0016052 | Biological Process |
| carbohydrate kinase activity | 2 | GO:0019200 | Molecular Function |
| NDP-glucose-starch glucosyltransferase activity | 1 | GO:0033840 | Molecular Function |
| starch grain | 1 | GO:0043036 | Cellular Component |
| chloroplast starch grain | 1 | GO:0009569 | Cellular Component |
| carbohydrate phosphatase activity | 1 | GO:0019203 | Molecular Function |
