# Supplementary figures and images for: Transcriptome analysis of carbohydrate metabolism during bulblet formation and development in Lilium davidii var. unicolor
Source: BMC Plant Biol. 2014 Dec 19;14:358. doi: 10.1186/s12870-014-0358-4 (PMC4302423; doi:10.1186/s12870-014-0358-4)

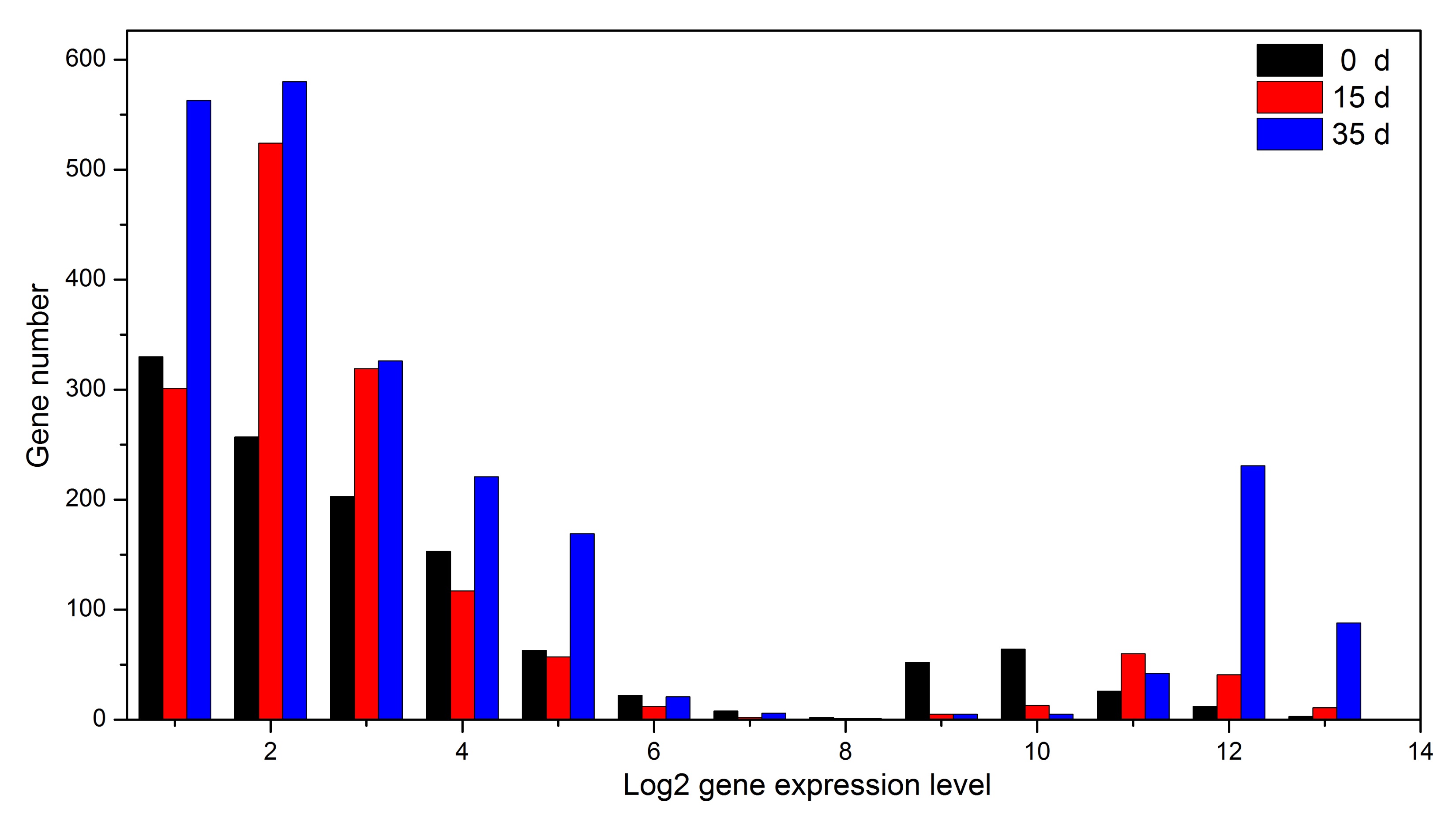

Supplement: Additional file 3: Figure S2. — Distribution of gene expression levels. [file 12870_2014_358_MOESM3_ESM.doc]

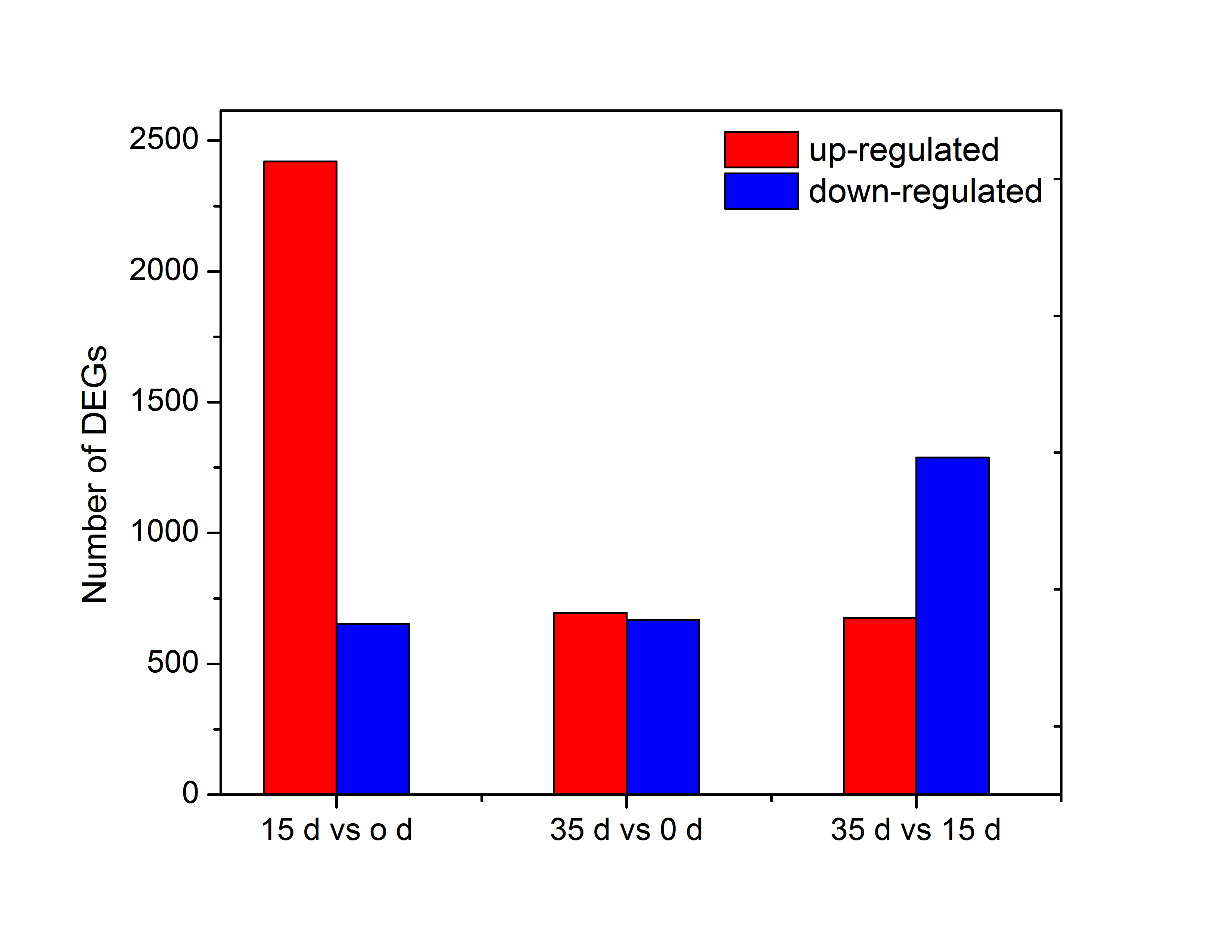

Supplement: Additional file 4: Figure S3. — Changes in gene expression profiles among different stages. [file 12870_2014_358_MOESM4_ESM.doc]

**M**

***SuSy1***

***SuSy2***

***SuSy3***

***INV1***


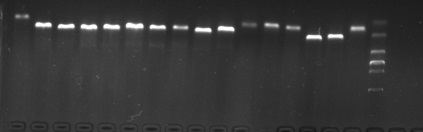


***INV2***

***SDBE1***

***SSS1***

***SSS2***

***SDBE2***

***SPS1***

***GBSS***

***SPS2***

***AGP1***

***AGP2***

***AGP3***

***SBE***

**500 bp**

**250 bp**

**100 bp**

Supplement: Additional file 7: Figure S4. — Gene specificity and amplicon size. Agarose gel (2%) electrophoresis showing amplification of a specific PCR product of expected size for each gene. M presents 2,000 bp DNA marker. [file 12870_2014_358_MOESM7_ESM.doc]
