# Supplementary material for: Transcriptome analysis of carbohydrate metabolism during bulblet formation and development in Lilium davidii var. unicolor
Source: BMC Plant Biol. 2014 Dec 19;14:358. doi: 10.1186/s12870-014-0358-4 (PMC4302423; doi:10.1186/s12870-014-0358-4)
Supplement: Additional file 5: Table S2. — List of first 15 pathways between library pairs. [file 12870_2014_358_MOESM5_ESM.doc]

| **Table S2** List of first 15 pathways between library pairs | | | |
| --- | --- | --- | --- |
| Pathway | DEGs with pathway annotation | All genes with pathway annotation | Pathway ID |
| *15 d vs 0 d* | | | |
| Ribosome | 76 | 545 | ko03011 |
| Chromosome | 64 | 672 | ko03036 |
| Plant-pathogen interaction | 33 | 178 | ko00360 |
| Chaperones and folding catalysis | 31 | 303 | ko03110 |
| Photosynthesis proteins | 30 | 114 | ko00194 |
| Spliceosome | 29 | 473 | ko03041 |
| Starch and sucrose metabolism | 28 | 193 | ko00500 |
| Plant hormone signal transduction | 24 | 239 | ko04075 |
| Transcription factors | 24 | 269 | ko03000 |
| Phenylpropanoid biosynthesis | 23 | 110 | ko00940 |
| Oxidative phosphorylation | 23 | 257 | ko00190 |
| Protein processing in endoplasmic reticulum | 22 | 275 | ko04141 |
| Ubiquitin system | 22 | 413 | ko04121 |
| Phenylalanine metabolism | 22 | 92 | ko00360 |
| Photosynthesis | 20 | 82 | ko00195 |
| *35 d vs 15 d* | | | |
| Ribosome | 53 | 545 | ko03011 |
| Chromosome | 33 | 672 | ko03036 |
| Plant-pathogen interaction | 26 | 178 | ko04626 |
| Phenylpropanoid biosynthesis | 23 | 110 | ko00940 |
| Phenylalanine metabolism | 21 | 92 | ko00360 |
| Chaperones and folding catalysts | 21 | 303 | ko03110 |
| Oxidative phosphorylation | 20 | 247 | ko00190 |
| Starch and sucrose metabolism | 18 | 193 | ko00500 |
| Protein processing in endoplasmic reticulum | 17 | 275 | ko05110 |
| Systemic lupus erythematosus | 17 | 100 | ko05322 |
| Proteasome | 16 | 160 | ko03051 |
| Plant hormone signal transduction | 15 | 239 | ko04075 |
| Ubiquitin system | 14 | 413 | ko04121 |
| Spliceosome | 14 | 473 | ko03041 |
| Cysteine and methionine metabolism | 14 | 105 | ko00270 |
| *35 d vs 0 d* | | | |
| Photosynthesis proteins | 29 | 114 | ko00194 |
| Chromosome | 29 | 672 | ko03036 |
| Photosynthesis | 19 | 82 | ko00195 |
| Transcription factors | 18 | 269 | ko03000 |
| Phenylpropanoid biosynthesis | 17 | 110 | ko00940 |
| Phenylalanine metabolism | 15 | 92 | ko00360 |
| Plant-pathogen interaction | 15 | 178 | ko04626 |
| Chaperones and folding catalysts | 15 | 303 | ko03110 |
| Methane metabolism | 14 | 140 | ko00680 |
| Glycolysis / gluconeogenesis | 14 | 180 | ko00010 |
| Carbon fixation in photosynthetic organisms | 13 | 122 | ko00710 |
| Starch and sucrose metabolism | 11 | 193 | ko00500 |
| Glutathione metabolism | 11 | 94 | ko00480 |
| Photosynthesis - antenna proteins | 10 | 31 | ko00196 |
| Cysteine and methionine metabolism | 10 | 105 | ko00270 |
