# Supplementary material for: Transcriptome analysis of carbohydrate metabolism during bulblet formation and development in Lilium davidii var. unicolor
Source: BMC Plant Biol. 2014 Dec 19;14:358. doi: 10.1186/s12870-014-0358-4 (PMC4302423; doi:10.1186/s12870-014-0358-4)
Supplement: Additional file 6: Table S3. — Expression of starch-sucrose metabolism genes of L. davidii var. unicolor. [file 12870_2014_358_MOESM6_ESM.doc]

| **Table S3**: Expression of starch-sucrose metabolism genes of *L. davidii* var. *unicolor* | | | | |
| --- | --- | --- | --- | --- |
| Unigene ID | Fold change  (0 d/15 d) | Fold change  (35 d/0 d) | Fold change  (35 d/15 d) | Annotation |
| lily_ck0_Unigene_BMK.12452 | 0.33 | 0.17 | 0.05 | 1,4-alpha-glucan-branching enzyme |
| lily_ck0_Unigene_BMK.1134 | 5.33 | 0.19 | 1.00 | acidic chitinase |
| lily_ck0_Unigene_BMK.26710 | 0.18 | 0.90 | 0.16 | ADPG pyrophosphorylase |
| lily_ck0_Unigene_BMK.14971 | 1.82 | 0.16 | 0.29 | all-*trans*-nonaprenyl-diphosphate synthase |
| lily_ck0_Unigene_BMK.2989 | 2.09 | 0.35 | 0.73 | alpha-amylase |
| lily_15_Unigene_BMK.28419 | 1.87 | 0.22 | 0.41 | alpha-glucan water dikinase |
| lily_35_Unigene_BMK.18519 | 0.12 | 2.13 | 0.25 | alpha-trehalose-phosphate synthase |
| lily_ck0_Unigene_BMK.1587 | 1.64 | 0.39 | 0.64 | alpha-trehalose-phosphate synthase |
| lily_15_Unigene_BMK.29880 | 0.45 | 0.53 | 0.24 | beta glucosidase |
| lily_35_Unigene_BMK.29350 | 1.86 | 0.15 | 0.29 | beta-1,3-glucanase |
| lily_15_Unigene_BMK.31286 | 0.22 | 0.50 | 0.11 | beta-amylase |
| lily_15_Unigene_BMK.34176 | 0.05 | 0.31 | 0.01 | beta-amylase |
| lily_35_Unigene_BMK.28820 | 0.87 | 2.40 | 2.08 | beta-amylase |
| lily_ck0_Unigene_BMK.12841 | 0.16 | 0.18 | 0.03 | beta-amylase |
| lily_ck0_Unigene_BMK.7227 | 2.63 | 0.26 | 0.69 | beta-galactosidase |
| lily_15_Unigene_BMK.31299 | 0.22 | 0.30 | 0.07 | beta-glucosidase |
| lily_35_Unigene_BMK.19333 | 1.64 | 0.14 | 0.23 | beta-glucosidase |
| lily_ck0_Unigene_BMK.4031 | 0.04 | 4.00 | 0.17 | beta-glucosidase |
| lily_35_Unigene_BMK.23176 | 0.04 | 23.00 | 1.00 | delta(24)-sterol reductase |
| lily_ck0_Unigene_BMK.10576 | 7.67 | 0.07 | 0.50 | endo-1,4-beta-mannosidase |
| lily_35_Unigene_BMK.2922 | 0.15 | 0.61 | 0.09 | fructokinase |
| lily_ck0_Unigene_BMK.24882 | 13.00 | 0.08 | 1.00 | fructose-1,6-bisphosphatase |
| lily_ck0_Unigene_BMK.10499 | 0.86 | 0.13 | 0.11 | galactinol-sucrose galactosyltransferase |
| lily_15_Unigene_BMK.29103 | 0.47 | 0.80 | 0.37 | glucose-1-phosphate adenylyltransferase |
| lily_ck0_Unigene_BMK.13755 | 0.17 | 1.00 | 0.17 | glucose-1-phosphate adenylyltransferase |
| lily_ck0_Unigene_BMK.24753 | 1.03 | 0.31 | 0.32 | glucose-1-phosphate adenylyltransferase |
| lily_15_Unigene_BMK.31079 | 0.12 | 1.00 | 0.12 | glucose-6-phosphate/phosphate translocator |
| lily_15_Unigene_BMK.25617 | 1.36 | 0.49 | 0.67 | granule-bound starch synthase |
| lily_ck0_Unigene_BMK.23134 | 0.59 | 0.38 | 0.22 | invertase |
| lily_15_Unigene_BMK.24803 | 12.50 | 0.04 | 0.50 | invertase |
| lily_15_Unigene_BMK.29998 | 0.01 | 9.00 | 0.13 | pectin methylesterase |
| lily_15_Unigene_BMK.30932 | 0.21 | 0.74 | 0.16 | pectinesterase |
| lily_ck0_Unigene_BMK.3330 | 2.10 | 0.16 | 0.33 | pectinesterase |
| lily_ck0_Unigene_BMK.7056 | 0.29 | 0.86 | 0.25 | pectinesterase |
| lily_ck0_Unigene_BMK.24221 | 2.46 | 0.55 | 1.37 | phosphoglucomutase |
| lily_35_Unigene_BMK.30073 | 0.23 | 5.77 | 1.32 | phosphorylase |
| lily_15_Unigene_BMK.25625 | 0.27 | 1.13 | 0.31 | polygalacturonase |
| lily_35_Unigene_BMK.6457 | 0.80 | 2.25 | 1.80 | protein phosphatase |
| lily_ck0_Unigene_BMK.418 | 1.00 | 8.00 | 8.00 | sedoheptulose-1,7-bisphosphatase |
| lily_15_Unigene_BMK.27248 | 0.67 | 0.47 | 0.31 | soluble starch synthase |
| lily_ck0_Unigene_BMK.27970 | 0.33 | 0.38 | 0.13 | soluble starch synthase |
| lily_15_Unigene_BMK.33308 | 3.00 | 0.33 | 1.00 | starch debranching enzyme |
| lily_ck0_Unigene_BMK.24998 | 5.00 | 0.13 | 0.67 | starch debranching enzyme |
| lily_35_Unigene_BMK.3485 | 4.25 | 0.53 | 2.25 | sucrose phosphate synthase |
| lily_ck0_Unigene_BMK.24435 | 7.50 | 0.40 | 3.00 | sucrose phosphate synthase |
| lily_15_Unigene_BMK.31406 | 2.49 | 0.49 | 1.22 | sucrose synthase |
| lily_35_Unigene_BMK.10649 | 0.47 | 0.60 | 0.28 | sucrose synthase |
| lily_35_Unigene_BMK.28655 | 4.00 | 0.59 | 2.38 | sucrose synthase |
| lily_15_Unigene_BMK.27673 | 1.07 | 0.18 | 0.19 | UDP-glucose 6-dehydrogenase |
| lily_ck0_Unigene_BMK.22945 | 0.17 | 0.65 | 0.11 | UDP-glucose 6-dehydrogenase |
| lily_15_Unigene_BMK.30188 | 5.34 | 0.31 | 1.66 | UDP-glucose pyrophosphorylase |
