# Supplementary material for: Transcriptome analysis of carbohydrate metabolism during bulblet formation and development in Lilium davidii var. unicolor
Source: BMC Plant Biol. 2014 Dec 19;14:358. doi: 10.1186/s12870-014-0358-4 (PMC4302423; doi:10.1186/s12870-014-0358-4)
Supplement: Additional file 8: Table S4. — Primers used in quantitative real-time PCR of L. davidii var. unicolor. [file 12870_2014_358_MOESM8_ESM.doc]

| **Table S4** Primers used in quantitative real-time PCR of *L. davidii* var. *unicolor* | | | | | | |
| --- | --- | --- | --- | --- | --- | --- |
| Unigene ID | Name | Annotation | Primer sequence (forward/reverse 5′-3′) | Amplicon length (bp) | PCR efficiency  (%) | Regression coefficient (R^2^) |
| lily_35_Unigene_BMK.10649 | *SuSy1* | sucrose synthase | F: TTTGAGGTGCTCGCAGGAAG  R: CTCAAAGTCCAATTCCAACACATAG | 173 | 95.3 | 0.9950 |
| lily_35_Unigene_BMK.28655 | *SuSy2* | sucrose synthase | F: CGCAGCGCCGACAATCTCTC  R: GTGGGAACGAGGCATTGAAGG | 272 | 96.7 | 0.9980 |
| lily_15_Unigene_BMK.31406 | *SuSy3* | sucrose synthase | F: CTGAAAGCTGCACAGGAAGCCATTG  R: AGGACGGGGAAAACTAGCATTGAAG | 282 | 101.3 | 0.9900 |
| lily_ck0_Unigene_BMK.24435 | *SPS1* | sucrose phosphate synthase | F: CCGATCTCTACAAGTCATGGGTCCG  R: TGCTGATTAGCCCAAGCCTCAAGAG | 127 | 94.2 | 0.9960 |
| lily_35_Unigene_BMK.3485 | *SPS2* | sucrose phosphate synthase | F: AGTTTACTATCCTGGCACTTCGC  R: AGTTTACTATCCTGGCACTTCGC | 134 | 98.7 | 0.9950 |
| lily_15_Unigene_BMK.24803 | *INV1* | vacuolar invertase | F: CTCGCCATAGGCTCTAAGGACCAGA  R: TTTGCAATTGGATCAAAGGACGCCT | 166 | 96.4 | 0.9930 |
| lily_ck0_Unigene_BMK.23134 | *INV2* | cell wall invertase | F: TTTGCAATTGGATCAAAGGACGCCT  R: TTCAACACATGCTTCACTCCCAAC | 122 | 104.5 | 0.9980 |
| lily_ck0_Unigene_BMK.13755 | *AGP1* | ADP-glucose pyrophosphorylase | F: TTAGAATCTGGTGTTGAACTGAAGG  R: TCGTCCTTGATGGTGGTGTTCTTAG | 130 | 103.9 | 0.9940 |
| lily_ck0_Unigene_BMK.24753 | *AGP2* | ADP-glucose pyrophosphorylase | F: TTGTTGATGGGAGCAGATTATTACG  R: AAATAGCCATCCGTCTCCCTTGC | 125 | 96.2 | 0.9980 |
| lily_15_Unigene_BMK.29103 | *AGP3* | ADP-glucose pyrophosphorylase | F: GGGAAGTGGGATTTGGGGTG  R: AACCGCAGGTGTGGCTCTTG | 116 | 105.4 | 0.9970 |
| lily_ck0_Unigene_BMK.12452 | *SBE* | starch branching enzyme | F: GAATGAGTAGTACGGAGCCCAAG  R: TATCCATAAGAACAAGCAAACCAAG | 96 | 98.3 | 0.9970 |
| lily_35_Unigene_BMK.27450 | *SDBE1* | starch debranching enzyme | F: GGCCTACTGGGTTGCGAAAAATCTC  R: TTTGAGTTACATTTTCTGGAAGC | 113 | 96.7 | 0.9980 |
| lily_15_Unigene_BMK.33308 | *SDBE2* | starch debranching enzyme | F: GACCAGTAATGAATCCCTGTTGAAG  R: TCATTGAAACTTCCGATGCCAG | 182 | 102.7 | 0.9990 |
| lily_15_Unigene_BMK.25617 | *GBSS* | granule bound starch synthase | F: GGGATGAACTTGGTGTATGTCG  R: TCTTATAGAGATGAAAGTAGCGCAC | 136 | 96.4 | 0.9960 |
| lily_15_Unigene_BMK.27248 | *SSS1* | soluble starch synthase | F: GTGGTCCTGTCGGCTTTATGTTC  R: TTGTGATCGGTCAGCTTCGC | 188 | 97.8 | 0.9950 |
| lily_ck0_Unigene_BMK.27970 | *SSS2* | soluble starch synthase | F: GAGTGGTATGGTGCTCTTGAGTGGG  R: TCTCCCAAGAGTAACCCTGGCTAAC | 128 | 95.3 | 0.9960 |
| lily_15_Unigene_BMK.29757 | *GAPDH* | glyceraldehyde-3- phosphate dehydrogenase | F: GCTGCAAGTTTCAACATTGTTCC  R: ATCATAAGTAGCCGCCTTCTCAA | 240 | 99.1 | 0.9970 |
